# The early surgical period in robotic radical hysterectomy is related to the recurrence after surgery in stage IB cervical cancer

**DOI:** 10.7150/ijms.59267

**Published:** 2021-05-13

**Authors:** Jiheum Paek, Peter C. Lim

**Affiliations:** 1 Division of Gynecologic Oncology, Department of Obstetrics and Gynecology, Ajou University School of Medicine, Suwon, Republic of Korea.; 2 Department of Gynecology Oncology and Robotic Surgery, Center of Hope, University of Nevada, Reno School of Medicine, Reno, NV, USA.

**Keywords:** cervical cancer, robotic surgery

## Abstract

**Objective:** To identify the pattern of recurrence and assess the clinicopathologic prognostic factors for survival after robotic radical hysterectomy (RRH) in the treatment of stage IB cervical cancer.

**Methods:** From December 2008 to March 2018, 64 cervical cancer patients who underwent RRH with pelvic lymph node dissection by a single surgeon were enrolled in this retrospective historical cohort timeline study. The patient's status was estimated in terms of operative outcomes, pathologic results, and survival outcomes.

**Results:** The median follow-up was 63 months. The recurrence rate was 9.4% (6/64). There were two recurrences at the vaginal vault, two in the pelvic cavity, and two at the peritoneum in the intraabdominal cavity. The overall survival rate was 95.3% (61/64). When patients were divided into three groups in order based on surgery date, the first surgical period showed significantly higher recurrence rate (21%) compared to both the second (10%) and the third period (0%) (p=0.037). Multivariate analysis showed that the early period of RRH (p=0.025) and clinical tumor size more than 3 cm (p=0.003) were prognostic factors related to the recurrence. Although there was no statistical significance, there has been no recurrence since a uterine manipulator was not used.

**Conclusion:** The early surgical period and large tumor were related to the disease recurrence after RRH. We suggest that the achievement of proficiency and appropriate patient selection are critical for prognosis after RRH in stage IB cervical cancer.

## Introduction

Cervical cancer is still one of the most common gynecologic malignancies although its incidence and mortality have decreased over the past 30 years in high-income countries 1-3. For treatment in patients with early-stage cervical cancer, radical hysterectomy has been standard treatment and minimally invasive surgery (MIS) as well as laparotomy has performed popularly with the evolution of the optimal instrumentation and surgical techniques. In 2018, the results from the Laparoscopic Approach to Cervical Cancer (LACC) trial, a randomized controlled trial, on surgery in early-stage cervical cancer showed that MIS had poorer survival outcomes compared to laparotomy 4. Since then, a great number of regarding studies have been reported and most of them have supported that MIS had poor survival outcome in cervical cancer patients 5-7. However, most of the evaluated patients who had MIS had laparoscopic radical hysterectomy (LRH), not robotic radical hysterectomy (RRH). Because robotic surgery, as it is known, has improved surgeons' dexterity and surgical precision 8,9, it has been performed popularly for complexed surgical procedures in deep and narrow pelvic cavity instead of laparoscopy or laparotomy in cervical cancer. Consequently, there have been several published studies that showed RRH was not inferior to LRH as well as open RH (ORH) 10-12. Therefore, it is needed to evaluate benefits and potential harms of robotic surgery in cervical cancer patients individually.

In this retrospective study, we evaluated the survival outcomes after RRH in patients with International Federation of Gynecology and Obstetrics (FIGO) 2009 stage IB cervical cancer. Additionally, we identified the pattern of recurrence and the clinicopathologic prognostic factors which may have contributed to survival.

## Materials and Methods

### Patients

This study was approved by the Institutional Review Board at University of Nevada, Reno, USA (1333119-1). We performed a retrospective study to determine pattern of recurrence and potential confounding factors which may have contributed to outcomes of RRH for treatment of cervical cancer. From December 2008 to March 2018, clinical FIGO 2009 stage IB cervical cancer patients who underwent RRH and pelvic lymph node dissection by a single surgeon were analyzed. We performed preoperative pelvic magnetic resonance imaging to determine clinical cancer stage, tumor size, and lymph node involvement. All analyzed patients were classified by the FIGO 2009 stage, not FIGO 2018 classification. The surgeon had performed 28 and 34 cases of ORH and LRH before starting robotic surgery, respectively. In addition, he started robotic surgery for endometrial cancer as well as cervical cancer at the almost same time. In the present study, we analyzed all patients who underwent RRH in the study period, including the first RRH case in 2008. Of these, patients who had stage IA, stage II, neuroendocrine and glassy cell type were excluded. The da Vinci Si or Xi Surgical System (Intuitive Surgical, Inc., CA, USA) was used. We used three robotic arms and one camera port. The instruments and accessories included Prograsp forcep, Monopolar spatula, Maryland bipolar forcep, Vessel sealer, and Mega suture-cut needle driver. A 12-mm trocar for the camera was inserted into the umbilicus. One 8-mm trocar for the left robotic arm and two 8-mm trocar for the right robotic arms were placed 8 cm apart bilaterally to the umbilicus. In addition, a 12-mm accessory assistant port was placed in the lower abdomen lateral to the left outer robotic port at the level of the anterior superior iliac spine. The surgical procedures of RRH were achieved in usual manner 13. The colpotomy was performed intracorporeally in all patients.

The patient's status was estimated in terms of operative outcomes, pathologic results, and survival outcomes. Operative outcomes included the types of RRH according to radicality 14, operating time, perioperative blood loss, days of hospitalization, and perioperative complications. In order to determine whether technique may have contributed to recurrences and survival, we evaluated the surgical periods and the utilization of a uterine manipulator. After all patients were arranged according to the order of surgery date, we divided them into three groups so that they had same total period. The early surgery period group (SP1) was from December 2008 to January 2012 (total period: 38 months). The total number of patients of the SP1 was 19 patients (7 patients in 2008, 6 in 2009, 2 in 2010, and 4 in 2011). The second SP group (SP2) was from February 2012 to February 2015 (total period: 37 months). The total number of patients of the SP2 was 20 patients (8 patients in 2012, 3 in 2013, and 9 in 2014). The late SP group (SP3) was from March 2015 to March 2018 (total period: 37 months). The total number of patients of the SP3 was 25 patients (8 patients in 2015, 9 in 2016, 6 in 2017, and 2 in 2018). The operating time was defined as the time from the first incision to the closure of the incision. For surgical complications, we used the Clavien-Dindo classification 15. Briefly, grade I included the deviation from the normal postoperative course without pharmacological treatment or surgical interventions. Grade II included the cases requiring blood transfusion as well as pharmacological treatment. Grade III included the cases requiring surgical intervention. Clinicopathologic factors that included clinical tumor size before surgery, tumor grade, lymphovascular space invasion, parametrium invasion, vaginal margin involvement, lymph node metastases, upstage after surgery, and postoperative adjuvant treatment, were evaluated for potential confounding factors which may have contributed to the recurrence and survival. The preoperative tumor size affects the clinical cancer stage and plays an important role in determining optimal treatment plan in newly diagnosed cervical cancer. Therefore, we distinguished the tumor size on imaging studies before surgery from pathologic tumor size. The postoperative adjuvant treatment was performed in patients who had high risk factor after RH, including lymph node metastases, parametrial involvement, and resection margin involvement.

### Statistical analysis

All continuous data were expressed as mean ± standard deviation, and categorical data were reported as an absolute number or percentage. Frequency distributions were compared using the Chi-square test and Fisher's exact test. Mean or median values were compared using the One-way ANOVA and Kruskal-Wallis tests. Progression-free survival (PFS) was calculated from the surgery date to date of disease progression or recurrence or date of last contact or disease-relevant death. Overall survival (OS) was calculated from the surgery date to date of last contact or death resulting from any cause. PFS and OS were estimated using the Kaplan-Meier method, and the differences in survival were compared using the log-rank test 16. The cox proportional hazard model was used for investigating the relationship between survival of patients and predictors 17. All calculated p values were two sided, and p<0.05 was considered statistically significant. Data were analyzed using SAS/STAT software, version 9.4 (SAS Institute Inc., NC, USA).

## Results

In the study period, 82 cervical cancer patients underwent RRH and 64 patients were enrolled in this retrospective study. A summary of subject clinicopathologic characteristics is described in Table 1. There was no difference among three SP groups. In addition, operative outcomes are shown in Table 2. At the earlier period (SP1), type C2 RRH was performed, and uterine manipulator was used for all patients. However, the performance of type C1 nerve-sparing RRH was increased more and more each period and uterine manipulator has not been used for the SP3. There was no clinically significant difference of operative outcomes among the three groups. Most of grade II complication included patients who required blood transfusion or suffered from urinary tract infection requiring antibiotics. The rate of grade III complication was 4.7% (3/64). One patient developed vesicovaginal fistula three months after completion of adjuvant radiotherapy. She underwent robotic vesicovaginal fistula repair six months after RRH. Two patients sustained dissectional cystotomy during RRH and underwent primary repair without any further complication. There was no significant difference of surgical complications among the three groups.

**Figure 1:**
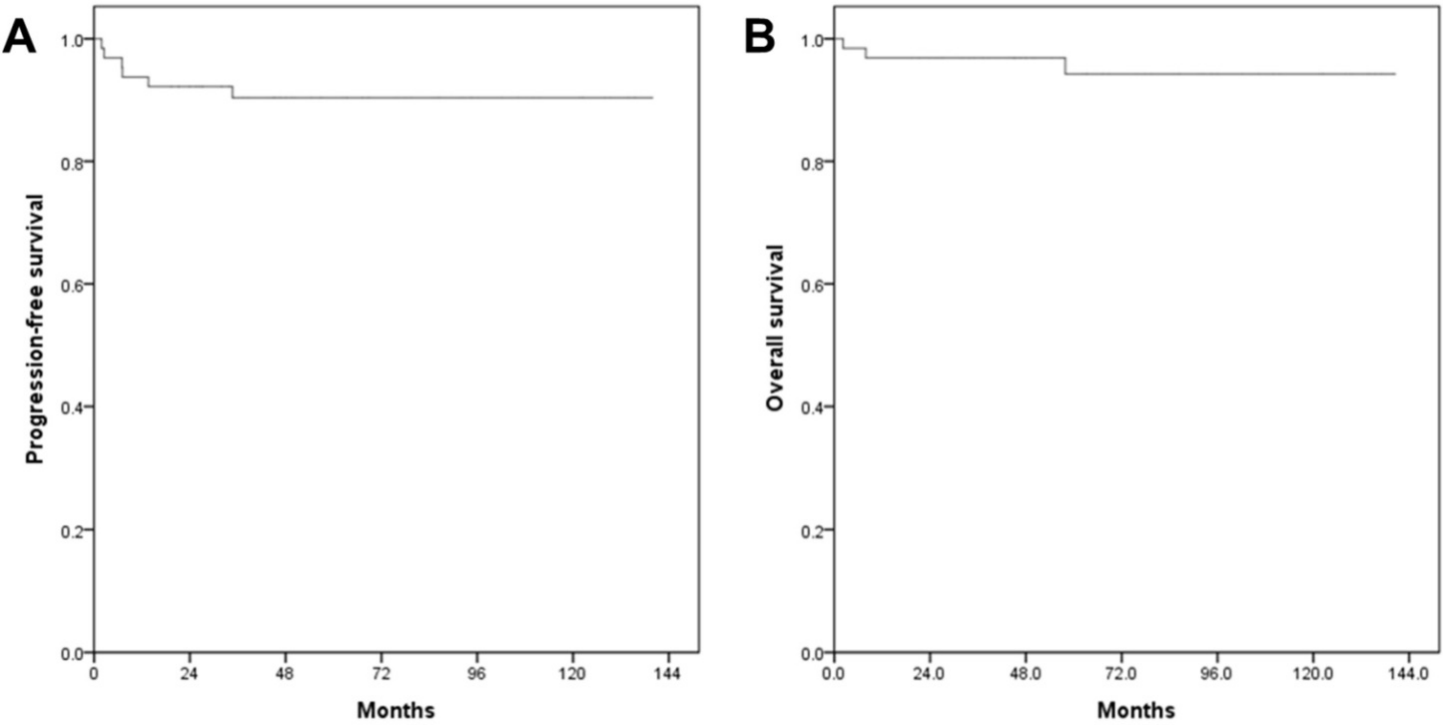
Survival outcomes in study population. All patients. (A) progression-free survival, (B) overall survival.

**Figure 2:**
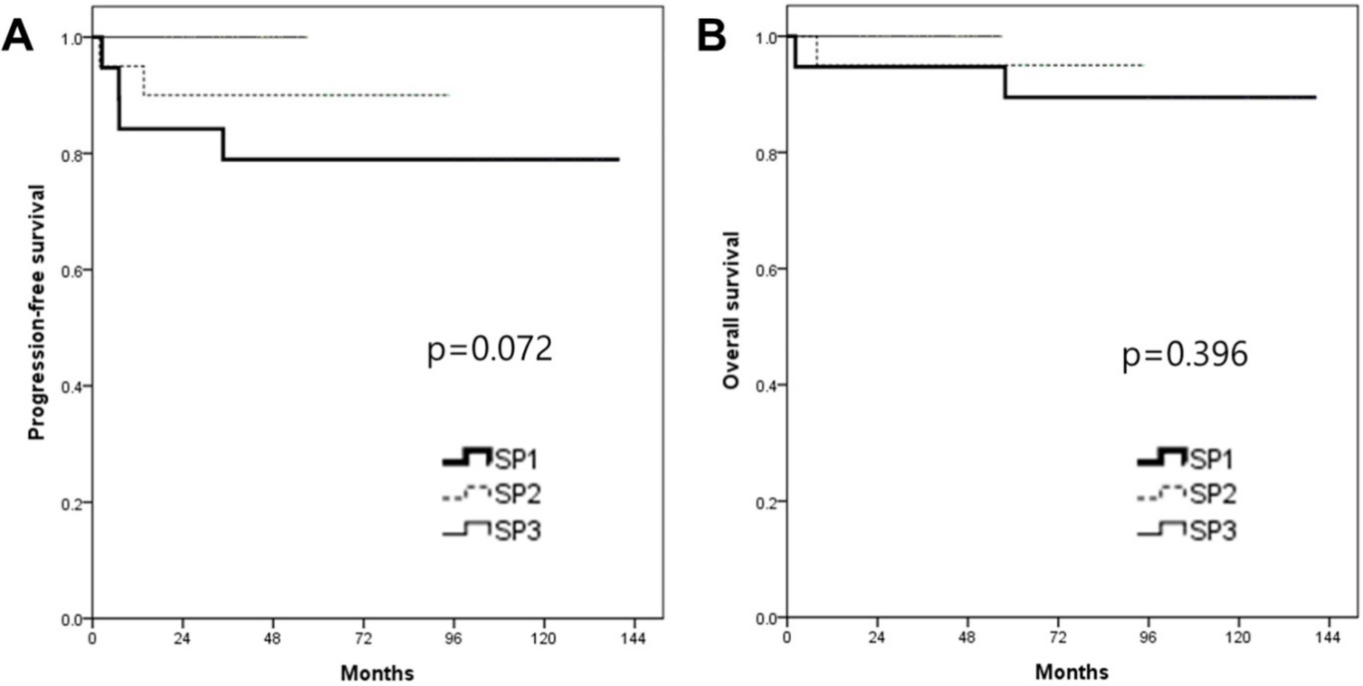
Kaplan-Meier analysis of survival outcomes according to the surgery period (SP). (A) progression-free survival and (B) overall survival among the 3 groups.

**Figure 3:**
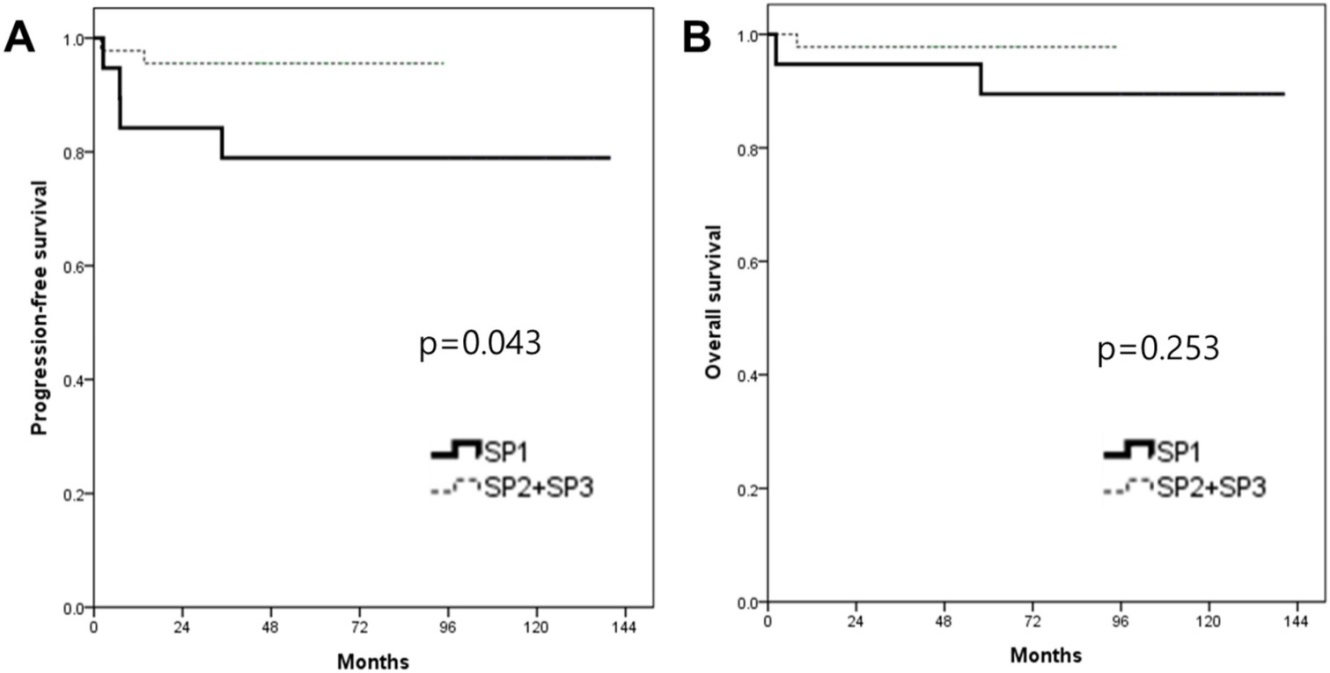
Kaplan-Meier analysis of survival outcomes according to the surgery period (SP). (A) progression-free survival, (B) overall survival between the 2 groups.

In the survival analysis, the median duration of the follow-up was 63.4 months (range 2.2-140.5). There was no patient who lost the follow-up. The mean PFS and OS of patients who underwent RRH were 66.9 and 71.7 months, respectively (Fig. 1). The recurrence rate was 9.4% (6/64). The overall survival rate was 95.3% (61/64). When patients were divided into three groups in order based on surgery date, the SP1 (2008-2011) showed significantly higher recurrence rate (21%, 4/19) compared to both the SP2 (2012-2014, 10%, 2/20) and the SP3 (2015-2018, 0%, 0/25) (p=0.037). Although there were no significant PFS differences among the three groups (p=0.072) (Fig. 2), the SP1 showed significantly poor PFS compared to both the SP2 and the SP3 (p=0.043) (Fig. 3). In addition, there were no significant OS differences among the groups.

Multivariate analysis showed that the early period of RRH (p=0.025, hazard ratio [HR] 26.70, 95% confidence interval [CI] 1.50-476.06) and clinical tumor size more than 3 cm (p=0.003, HR 87.25, 95% CI 4.58-1663.78) were prognostic factor related to the recurrence (Table 3). Although there was no statistical significance, there has been no recurrence since use of a uterine manipulator was discontinued for last 25 patients. There was no predictor related to OS via multivariate analysis. Table 4 shows a summary of all six patients who had recurrence. Of these 6 patients, there were two recurrences at the vaginal vault, two in the pelvic cavity, and two at the peritoneum in the intraabdominal cavity.

## Discussion

The LACC trial, a prospective randomized control study, provided level I evidence that PFS and OS was better for ORH compared to MIS RH for treatment of early-stage cervical cancer 4. Similarly, a large National Cancer Data Base and the Surveillance, Epidemiology, and End Results trial also reported MIS was associated with increased probability of deaths (9.1 vs. 5.3%) within 4 years compared to ORH. In their subgroup analysis of tumor size < 2 cm, however, there was no difference in survival between the two surgical groups 18. Likewise, a retrospective case match control study from Korea reported LRH had poorer PFS compared to ORH (85.4 vs. 91.8%) 19. In their subgroup analysis regarding tumor size < 2 cm, there was no difference in outcome between the two groups. In addition, a population-based study from Canada reported poorer survival outcome in MIS RH compared to ORH for treatment of early-stage cervical cancer 20. In contradistinction, a nationwide population-based cohort study in Sweden compared OS and PFS of RRH with ORH for early-stage cervical cancer 21. The 5-year OS was 92% and 94% and PFS was 84% and 88% for the open and robotic groups, respectively. There was neither a difference of survival nor recurrence pattern between the two groups.

The limitation of these studies and LACC trial is that it was difficult to ascertain whether the potential confounding factors, including surgeon's learning curve, surgical volume, utilization of a uterine manipulator, intraoperative tumor spillage, and subsequent aerosolization, contributed the observed inferior outcome. In the LACC trial, 84% of patients enrolled in MIS group underwent laparoscopic and only 16% did the robotic approach. Although some of above-mentioned studies observed similar poor outcomes with larger cohort for robotic groups, they were in early phase of robotic surgical adoption. Therefore, it might be overlooked that the surgical period or learning curve of robotic surgery could be a potential contributing factor for poor outcomes observed in MIS groups. In addition, different surgical procedures among surgical approaches, including parametrial dissection, the use of a uterine manipulator, and the route for colpotomy, were not considered as possible confounding factors for recurrence and survival.

In the present study, we performed a retrospective timeline cohort study of a single surgeon's surgical experience. Based on the chronological surgery date, the first period (SP1) showed significantly higher recurrence rate (21%) compared to both the second (SP2) and the third period (SP3) (p=0.037). The last patient experienced recurrence in 2014. Immature follow-up data may be a possible explanation for no recurrence in the SP3 (2015-2018). However, the median follow-up in this subgroup cohort was 48.7 months (range 23.3-61.1 months). Compared that the PFS in the SP1 (2008-2012) was ranged from 2.5 to 34.7 months, we considered that the SP3 also had sufficient time for follow-up. Similarly, a Dutch group reported that at least 61 cases were needed to get the proficiency of RRH in cervical cancer and both PFS and OS significantly increased after the learning period 22. In addition, several studies also reported the institutional surgical experience was one of the most important factors related to the survival outcomes after RRH 23,24. With accumulated experiences, surgeons will be able to get proper radicality during the RH and to acquire surgical techniques to minimize the spillage of cancer tissues. Moreover, it has been only fifteen years since robotic surgery has been performed in cervical cancer. Surgeons need to acquire new robotic surgical techniques in order to reproduce the procedures of ORH or LRH. Therefore, the notion of learning curve in adopting a complex surgical procedure, including the RH, should be considered when we evaluate oncological outcome after surgery. In addition, we performed only type C2 RRH in the early surgical period (SP1). In literature, it was 2011 that the feasibility and techniques of type C1 nerve-sparing RRH was reported firstly by Magrina et al. 25. After there have been several studies that showed the type C1 RRH decreased urinary dysfunction after surgery without the compromise of survival outcomes 26,27, we have started implementing it gradually in our institution.

Little is known about the relationship between surgical volume and oncologic outcome. Matsuo et al examined the association between surgical volume and survival in patients with early-stage cervical cancer who underwent RH 28. The surgical volume per institution was defined as low (< 32 surgeries), mid, and high-volume (≥ 105 surgeries). Their multivariate analysis showed that high-volume institutions had a lower risk of recurrence though their analysis was confined to ORH (HR 0.69, p<0.001). Doo et al reported that RRH and tumor size ≥ 2 cm were significantly related to poor survival in a single high surgical volume institution 29. However, this study analyzed a total of 49 patients for six years. It is unclear how many cases are needed to be considered as high surgical volume or to get proficiency in terms of RRH. However, it is obvious that both surgical consistency and repetition of surgical performance with regular surgical volume may be critical contributing factors to surgical and oncological outcomes.

The other potential confounding factor is peritoneal dissemination by intraoperative tumor spillage or excessive manipulation of tumor tissues 30,31. We theorized that the use of a uterine manipulator during RRH might result in tumor spillage into the peritoneal cavity and combination of steep Trendelenburg position and aerosolization might have facilitated the intraabdominal seeding which leads to worse oncological outcome. Kanao et al evaluated survival outcomes of LRH without uterine manipulator and intracorporeal colpotomy by comparison with ORH and there was no difference of PFS and OS between the two groups 32. In our experience, the use of a uterine manipulator showed marginal significance for contributing factor of PFS in the univariate analysis (p=0.074). However, this difference was not observed in multivariate analysis. The reason might be that the confounding variables which were analyzed in multivariate analysis, including surgery period and use of a uterine manipulator, interrupted one another as a time factor. If there is no statistical significance, it does not mean that its possibility as a contributing factor for survival outcomes can be ignored. We have eliminated a uterine manipulator during RRH since 2015. Since then, there was no recurrence in all 25 patients who underwent RRH without it. On the other hand, all recurrences occurred in the group which used a uterine manipulator (6/39, 15.4%). Out of 6 recurred patients, there were two recurrences at the vaginal vault, two in the pelvic cavity, and two at the peritoneum in the intraabdominal cavity. The two patients who had recurrence at the intraabdominal peritoneum died. The observed recurrence pattern was unusual compared to the pattern after ORH which generally showed recurrence at the vaginal apex or lymph nodes. Additionally, Kong et al reported that the intracorporeal colpotomy group had inferior PFS and higher peritoneal recurrence compared to vaginal colpotomy in patients who underwent LRH or RRH 33,34. We could not assess the route of colpotomy as a contributing factor for PFS because the colpotomy was performed intracorporeally in all patients in our study. We propose that vaginal occlusion should be considered prior to colpotomy to minimize spillage of tumor cell in addition to the disuse of a uterine manipulator. Further studies should be performed to assess the role of the route of colpotomy and uterine manipulator as prognostic factors after RRH.

Limitations of this study were that this was a retrospective study without the comparison with other surgical approaches and that we were unable to assess surgical volume as a potential contributing factor to the observed oncological outcome. Secondly, we divided the patients into three groups so that they had same total period without the cut-off values for division. Consequently, we could not avoid the statistical bias completely. In spite of the small number of enrolled patients, we could analyze survival outcomes after RRH clearly because we focused on only stage IB patients. Additionally, we evaluated a single surgeon experience of RRH for treatment of cervical cancer and the oncological outcomes with long-term follow-up. A single surgeon's experience afforded us the ability to evaluate the potential confounding factors with surgical consistency.

In conclusions, the early surgical period and tumor size were related to the disease recurrence after RRH. If we consider RRH as a treatment option, appropriate patient selection, eliminating use of a uterine manipulator to minimize potential cancer cells seeding into the peritoneal cavity, and achievement of proficiency are critical for prognosis after RRH in stage IB cervical cancer.

## Figures and Tables

**Table 1:** Clinicopathologic characteristics

	Overall (n = 64)	SP1 (n = 19)	SP2 (n = 20)	SP3 (n = 25)	p value
Age (years ± SD)	45.1 ± 11.6	45.1 ± 13.9	46.8 ± 11.4	43.8 ± 10.1	0.699
Body mass index (kg/m^2^ ± SD)	26.7 ± 6.1	27.2 ± 7.3	25.2 ± 5.0	27.4 ± 5.9	0.418
**Tumor stage (%)**					0.391
IB1	55 (85.9)	18 (94.7)	16 (80.0)	21 (84.0)	
IB2	9 (14.1)	1 (5.3)	4 (20.0)	4 (16.0)	
Tumor size (cm, IQR)	1.5 (2)	1 (1)	1.5 (2)	2 (2)	0.454
**Histology (%)**					0.199
Squamous cell carcinoma	32 (50)	11 (57.9)	12 (60.0)	9 (36.0)	
Adenocarcinoma	32 (50)	8 (42.1)	8 (40.0)	8 (64.0)	
**Tumor grade (%)**					0.485
Well differentiated	20 (31.3)	6 (31.6)	4 (20.0)	10 (40.0)	
Moderately differentiated	30 (46.9)	8 (42.1)	10 (50.0)	12 (48.0)	
Poorly differentiated	14 (21.9)	5 (26.3)	6 (30.0)	3 (12.0)	
Lymphovascular space invasion (%)	15 (26.8)	5 (26.3)	6 (30.0)	4 (16.0)	0.360
Parametrium invasion (%)	3 (4.7)	2 (10.5)	1 (5.0)	0	0.261
Lymph node metastases (%)	12 (18.8)	4 (21.1)	4 (20.0)	4 (16.0)	0.900
Vaginal cuff margin involvement (%)	1 (1.6)	0	0	1 (4.0)	0.453
Postoperative adjuvant treatment (%)	13 (20.3)	5 (26.3)	4 (20.0)	4 (20.0)	0.701

SP, surgical period; SD, standard deviation; IQR, interquartile range.

**Table 2:** Operative outcomes

	Overall (n = 64)	SP1 (n = 19)	SP2 (n = 20)	SP3 (n = 25)	p value
**Type of radical hysterectomy (%)**					< 0.0001
C1 (nerve-sparing)	34 (53.1)	0	12 (60.0)	22 (88.0)	
C2	30 (46.9)	19 (100.0)	8 (40.0)	3 (12.0)	
**Use of uterine manipulator (%)**					< 0.0001
Yes	39 (60.9)	19 (100.0)	20 (100.0)	0	
No	25 (39.1)	0	0	25 (100.0)	
Operating time (min, ± SD)	201.3 ± 58.4	212.4 ± 65.3	182.2 ± 39.6	208.6 ± 64.3	0.206
Estimated blood loss (ml, IQR)	100 (113)	100 (100)	100 (88)	100 (63)	0.097
Postoperative hemoglobin drop (g/dl ± SD)	2.2 ± 1.0	2.2 ± 1.2	2.3 ± 1.0	2.1 ± 0.7	0.895
Days of hospitalization (IQR)	1 (1)	1 (2)	1 (2)	1 (1)	0.037
Number of lymph nodes retrieved (± SD)	27.2 ± 10.1	32.2 ± 8.7	24.4 ± 11.0	24.9 ± 8.9	0.020
**Perioperative complications (%)**					
Grade I	3 (4.7)	2 (10.5)	1 (5.0)	0	0.261
Grade II	14 (21.9)	6 (31.6)	4 (20.0)	4 (20.0)	0.504
Grade III	3 (4.7)	2 (10.5)	1 (5.0)	0	0.261

SP, surgical period; SD, standard deviation; IQR, interquartile range.

**Table 3:** Univariate and multivariate analyses for prognostic factor of recurrence

	Univariate analysis	Multivariate analysis	
	p value	p value	HR (95% CI)
Age ≥ 50 years	0.649		
Nerve-sparing RRH	0.090	0.733	0.61 (0.04-10.08)
Use of uterine manipulator	0.074		
Early period of surgery (SP1)	0.037	0.025	26.70 (1.50-476.06)
Clinical tumor size ≥ 2 cm	0.099		
Clinical tumor size ≥ 3 cm	0.007	0.003	87.25 (4.58-1663.78)
Tumor stage IB2	0.847		
Upstage after surgery	0.460		
Tumor grade 3	0.113	0.999	1.00 (0.17-6.04)
Lymphovascular space invasion	0.289		
Parametrium involvement	0.568		
Lymph nodes involvement	0.891		
Vaginal cuff margin positive	0.746		
Postoperative adjuvant treatment	0.816		

HR, hazard ratio; CI, confidence interval; RRH, robotic radical hysterectomy; SP, surgical period.

**Table 4:** Clinicopathologic characteristics of patients with recurrence

Patients	1	2	3	4	5	6
Sites of recurrence	pelvic cavity	pelvic cavity	intraabdominal	vaginal vault	intraabdominal	vaginal vault
Recurrence (months)	7.1	2.5	34.7	7	1.9	13.6
Death (months)	-	-	+ (57.9)	-	+ (7.9)	-
No. of RRH (1~64)	#1	#3	#10	#19	#24	#37
Surgical period (1~3)	1	1	1	1	2	2
Use of uterine manipulator	+	+	+	+	+	+
Radical type	C2	C2	C2	C2	C2	C1
Age (years)	33	38	47	60	31	49
Tumor stage	IB1	IB2	IB1	IB1	IB1	IB1
Histology	SCC	SCC	AC	AC	SCC	AC
Tumor size (cm)	3	4	1	3	3	3
Tumor grade (1~3)	3	3	1	2	2	3
Lymphovascular space invasion	+	N/A	N/A	-	-	+
Parametrial invasion	-	-	-	-	-	-
Lymph node metastases	-	+	-	-	-	-
Vaginal cuff margin involvement	-	-	-	-	-	-
Adjuvant treatment	-	+ (CCRT)	-	-	-	-

RRH, robotic radical hysterectomy; SCC, squamous cell carcinoma; AC, adenocarcinoma; N/A, non-available; CCRT, concurrent chemo-radiation therapy.
